# Evaluating the OpenAI’s GPT-3.5 Turbo’s performance in extracting information from scientific articles on diabetic retinopathy

**DOI:** 10.1186/s13643-024-02523-2

**Published:** 2024-05-16

**Authors:** Celeste Ci Ying Gue, Noorul Dharajath Abdul Rahim, William Rojas-Carabali, Rupesh Agrawal, Palvannan RK, John Abisheganaden, Wan Fen Yip

**Affiliations:** 1 Health Services and Outcomes Research, National Healthcare Group, 3 Fusionopolis Link, #03-08, Nexus@One-North, Singapore, 138543 Singapore; 2 National Healthcare Group Eye Institute, Tan Tock Seng Hospital, 11 Jalan Tan Tock Seng, Singapore, 308433 Singapore; 3 Lee Kong Chian School of Medicine, Nanyang Technological University, 11 Mandalay Road, Singapore, 308232 Singapore

**Keywords:** Information extraction, Concordance, GPT-3.5 Turbo

## Abstract

We aimed to compare the concordance of information extracted and the time taken between a large language model (OpenAI’s GPT-3.5 Turbo via API) against conventional human extraction methods in retrieving information from scientific articles on diabetic retinopathy (DR). The extraction was done using GPT3.5 Turbo as of October 2023. OpenAI’s GPT-3.5 Turbo significantly reduced the time taken for extraction. Concordance was highest at 100% for the extraction of the country of study, 64.7% for significant risk factors of DR, 47.1% for exclusion and inclusion criteria, and lastly 41.2% for odds ratio (OR) and 95% confidence interval (CI). The concordance levels seemed to indicate the complexity associated with each prompt. This suggests that OpenAI’s GPT-3.5 Turbo may be adopted to extract simple information that is easily located in the text, leaving more complex information to be extracted by the researcher. It is crucial to note that the foundation model is constantly improving significantly with new versions being released quickly. Subsequent work can focus on retrieval-augmented generation (RAG), embedding, chunking PDF into useful sections, and prompting to improve the accuracy of extraction.

## Introduction

It is critical for policy and clinical guidelines to be based on the best available evidence [5]. Systematic reviews are often regarded as the gold standard for evidence synthesis [8], appraising the latest evidence transparently and objectively by employing established standards [2]. Consequently, poorly conducted systematic reviews can potentially misinform future clinical guidelines and policies [8]. Misinformed clinical guidelines could equip practitioners with inaccurate scientific information and clinical advice, compromising the quality of care [9]. Additionally, misinformed clinical guidelines could potentially encourage ineffective, harmful, or wasteful interventions [9].

In recent years, as the number of primary studies continues to increase, current methods of manual information extraction by researchers for the synthesis of systematic reviews is not sustainable and efficient. Depending on the experience of the researcher and the number of studies selected, conducting a systematic review can take up to 2 years [7]. However, given the surge of artificial intelligence (AI) in recent years, there is potential for it to be a powerful tool to speed up the process of information extraction from each scientific article. In particular, large language models (LLMs), a generative AI, such as the generative pre-trained transformer (GPT), which can process and generate text, may be adopted to expedite the systematic review process. Layering upon LLMs, the retrieval-augmented generation process (RAG), an information retrieval component, can improve the accuracy of information extraction of articles, delivering more precise and contextually relevant responses [3].

Systematic reviews require high accuracy in methods, which may be difficult for AI to attain [6]. One of the challenges of accuracy is the occurrence of potential hallucination, where it produces information that may sound plausible but are either factually incorrect or unrelated to the given context [10]. As such, evaluating the performance of AI tools adopted for this process is critical. In recent times, there has been a lot of research evaluating the performance of an LLM that is open for public use, ChatGPT, in medical research [1]. The accuracy of ChatGPT in answering medical queries regarding different domains such as cancer, liver diseases, and COVID-19 vaccination, has been assessed [1]. The results reported different accuracy ranges of ChatGPT, from 18.3 to 100% [1]. However, the performance of OpenAI’s GPT-3.5 Turbo in extracting information from scientific articles stored in PDF format has not been evaluated.

This study aimed to compare the concordance of information extracted and the time taken between OpenAI’s GPT-3.5 Turbo against conventional human extraction methods in retrieving relevant information from scientific articles on diabetic retinopathy (DR).

## Methods

Twenty papers on diabetic retinopathy (DR) were randomly selected from PubMed. Information on (1) country of study, (2) significant risk factors of DR, (3) inclusion and exclusion criteria, and (4) odds ratio (OR) and 95% confidence interval (CI) was extracted by the first researcher. A second researcher checked the information extracted by the first researcher. Discrepancies between the two researchers were resolved through discussions with a third researcher.

**Fig. 1:**
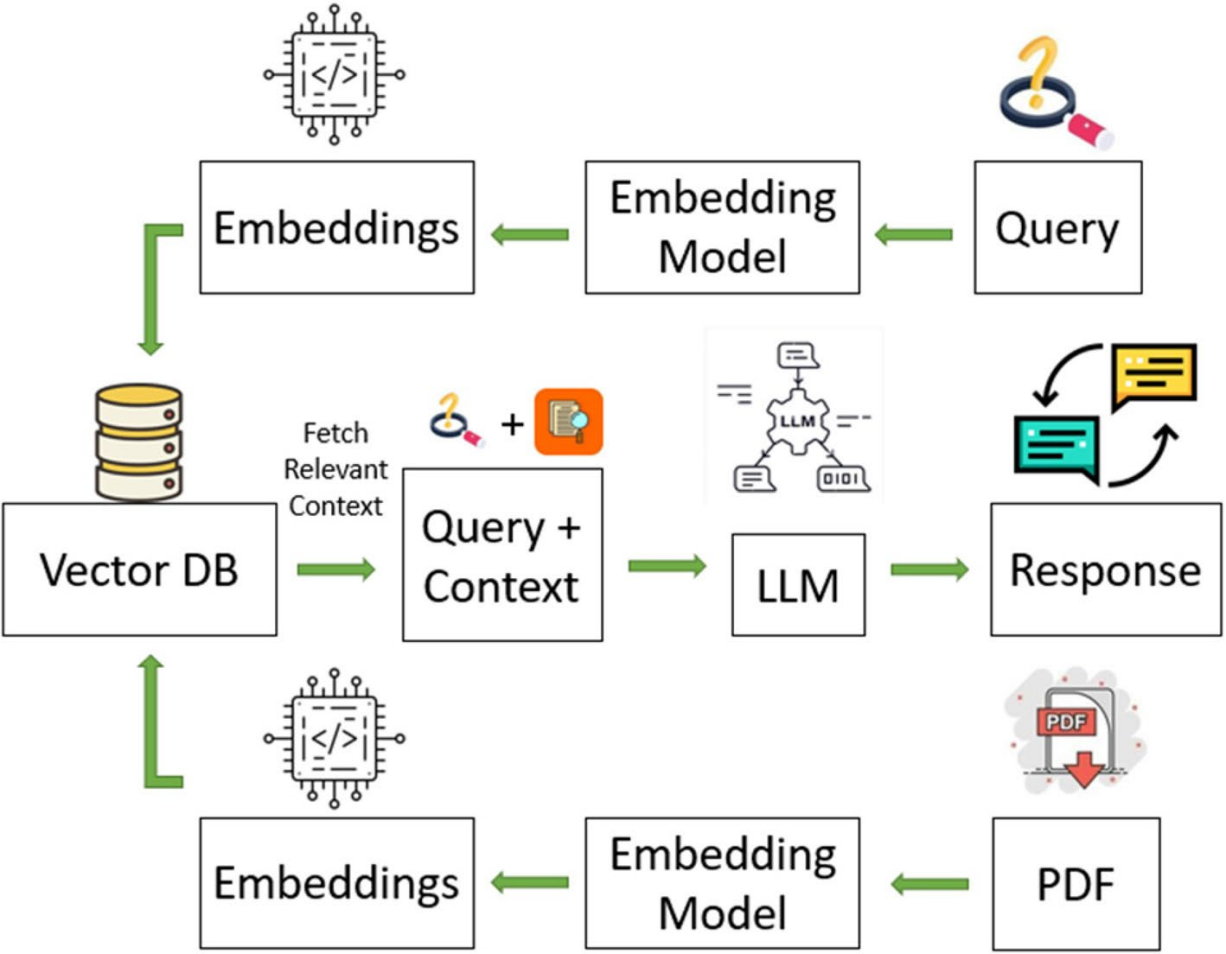
Retrieval-augmented generation of OpenAI

Using the OpenAI application programming interface (API), we invoked a question and answer (QA) model using GPT-3.5 Turbo, as of October 2023. The twenty papers were processed by OpenAI’s GPT-3.5 Turbo as an entire batch of PDF files. Instructional prompts were used to query the same information from all articles using OpenAI’s GPT-3.5 Turbo (Fig. 1). A complete match between the 2 approaches was defined as accurate. Concordance for each information extraction was calculated as the number of articles with accurate information extracted by OpenAI’s GPT-3.5 Turbo divided by the total number of articles. The time taken for extraction was also assessed.

## Results

Of the twenty papers, GPT-3.5 Turbo was unable to extract information from three (15%) articles as they were not in PDF format. For the remaining seventeen (85%) papers, GPT-3.5 Turbo took 5 min compared to 1310 min by the researcher. Concordance between GPT-3.5 Turbo and manual extraction by the researcher was highest for the extraction of the country of study at 100%, 64.7%% for significant risk factors of DR, 47.1% for inclusion and exclusion criteria, and 41.2% for OR and 95% CI (Table 1). The concordance levels seem to indicate the complexity associated with each prompt.

**Table 1:** Concordance of OpenAI’s GPT-3.5 Turbo in the information extracted

	Country	Significant risk factor	Exclusion and inclusion criteria	Odds ratio and 95% CI
No. of articles with accurate information extracted	17	11	8	7
Concordance (%)	100	64.7	47.1	41.2

## Discussion

OpenAI’s GPT-3.5 Turbo performance in extracting specific information varied from excellent to poor. The availability of required information in the main text and the complexity of prompts may be potential reasons to explain the poor concordance observed for information extraction of (1) odds ratio and 95% CI and (2) inclusion and exclusion criteria. Specifically, complete information on odds ratio and 95% CI are often presented as images, which cannot be extracted by OpenAI’s GPT-3.5 Turbo. It was also observed that the performance of GPT3.5 Turbo decreases when each prompt contains two different types of information to be extracted. Nonetheless, despite the limited performance of GPT-3.5 Turbo, our results demonstrated that the use of GPT-3.5 Turbo had significantly reduced the time taken for information extraction. This will improve productivity, with the use of the AI as an assistant.

GPT-3.5 Turbo performance in information extraction is dependent on the availability of required information in the main text and the complexity of prompts. Information extraction for systematic review is a mammoth task, with large amounts of information to be extracted from many articles. GPT-3.5 Turbo may be adopted to extract simple information that can be easily found in the text (e.g., country of study), leaving more complex information (e.g., odds ratio, 95% CI) to be extracted by the researcher. Researchers should continue to review the information extracted by GPT-3.5 Turbo. Adoption of GPT-3.5 Turbo into the process will expedite the process of information extraction and reduce researchers’ fatigue. To the best of our knowledge, there are no published studies available that evaluated the concordance of information extracted from scientific articles with conventional human extraction methods. However, there is a study that examined the performance of GPT-3.5 Turbo for abstract screening. In that study, the authors reported that precise and detailed prompts yielded more accurate responses [4]. The authors also highlighted that GPT should be engaged as an assistant to compliment the screening process to enhance the speed of the process. The authors’ findings are similar to what was observed in our study.

Future applied research could also focus on RAG, embedding, chunking PDF into useful sections, and prompting to improve the accuracy of extraction. Additionally, it is crucial to note that the foundation model of the large language model, GPT, is constantly improving significantly with new versions being released quickly. With the rapid evolvement of LLMs, robust evaluation frameworks will be needed to (1) continuously provide feedback and (2) evaluate and monitor the performance, to support information extraction for systematic reviews.

## Conclusion

Our results suggest that OpenAI’s GPT-3.5 Turbo may be adopted to extract simple information that is easily located in the text. However, human researchers are still needed to do the task of more complex information that may be presented in the tables or images of the articles.

## Data Availability

Data arising from the review may be made available from the corresponding author upon reasonable request.

## Abbreviations

DR: Diabetic retinopathy
AI: Artificial intelligence
LLM: Large language model
GPT: Generative pre-trained transformer
OR: Odds ratio
CI: Confidence interval
RAG: Retrieval-augmented generation
API: Application programming interface

## References

[1] Bagde H, Dhopte A, Alam MK, Basri R (2023). A systematic review and meta-analysis on ChatGPT and its utilization in medical and dental research. Heliyon.

[2] Haddaway NR, Pullin AS (2014). The policy role of systematic reviews: past, present and future. Springer Sci Rev.

[3] Innovation, S. (2023, November 9). Implementing a retrieval-augmented generation (RAG) system with OpenAI’s API using LangChain. *Medium*. https://scalexi.medium.com/implementing-a-retrieval-augmented-generation-rag-system-with-openais-api-using-langchain-ab39b60b4d9f

[4] Kohandel Gargari O, Mahmoudi MH, Hajisafarali M, Samiee R (2024). Enhancing title and abstract screening for systematic reviews with GPT-3.5 turbo. BMJ Evid-Based Med.

[5] Mancin S, Sguanci M, Andreoli D, Soekeland F, Anastasi G, Piredda M, De Marinis MG (2024). Systematic review of clinical practice guidelines and systematic reviews: a method for conducting comprehensive analysis. MethodsX.

[6] Marshall IJ, Wallace BC (2019). Toward systematic review automation: a practical guide to using machine learning tools in research synthesis. Syst Rev.

[7] Nussbaumer-Streit B, Ellen M, Klerings I, Sfetcu R, Riva N, Mahmić-Kaknjo M, Poulentzas G, Martinez P, Baladia E, Ziganshina LE, Marqués ME, Aguilar L, Kassianos AP, Frampton G, Silva AG, Affengruber L, Spjker R, Thomas J, Berg RC, … Gartlehner G. Resource use during systematic review production varies widely: a scoping review. J Clin Epidemiol 2021;139:287–296. 10.1016/j.jclinepi.2021.05.019 10.1016/j.jclinepi.2021.05.019 34091021

[8] Uttley L, Quintana DS, Montgomery P, Carroll C, Page MJ, Falzon L, Sutton A, Moher D (2023). The problems with systematic reviews: a living systematic review. J Clin Epidemiol.

[9] Woolf SH, Grol R, Hutchinson A, Eccles M, Grimshaw J (1999). Potential benefits, limitations, and harms of clinical guidelines. BMJ : Brit Med J.

[10] Zhou C, Neubig G, Gu J, Diab M, Guzman P, Zettlemoyer L, Ghazvininejad M. Detecting hallucinated content in conditional neural sequence generation (arXiv:2011.02593). arXiv. 2021. 10.48550/arXiv.2011.02593

